# The implementation of a Cardiac Scientist Led Heart Failure diagnosis clinic for non-urgent suspected heart failure referrals

**DOI:** 10.1186/s44156-025-00098-9

**Published:** 2025-12-15

**Authors:** Jonathan Cook, Jin Jiang, Ayisha Khan-Kheil, Thomas E. Ingram

**Affiliations:** Royal Wolverhampton NHS Foundation Trust, New Cross Hospital, Wolverhampton Road, Wolverhampton, West Midlands WV10 0QP UK

**Keywords:** Heart failure, Echocardiography, Scientist led

## Abstract

**Background:**

NICE guidelines suggest that patients with suspected heart failure (HF) and an NT-proBNP between 400-2,000ng/l have specialist assessment and transthoracic echocardiography within 6 weeks. Typically, pathways require significant coordination of a multidisciplinary team, with upfront input from senior clinicians. In line with NHS workforce plans to develop non-medical roles, a Cardiac scientist led Heart Failure assessment clinic for non-urgent suspected HF referrals was trialled. A British Society of Echocardiography accredited physiologist with health assessment and clinical examination qualifications performed a transthoracic echocardiogram, 12 lead electrocardiogram and clinical assessment within one appointment slot. Preliminary outcome recommendations made by the physiologist were reviewed at a later point by a supervising HF consultant. The pilot aimed to ensure timely clinical assessment at the point of echocardiography and reduce delays in patient diagnosis. In a retrospective service evaluation, the clinic decision and time from scan to outcome correspondence was compared between the cardiac clinical scientist led pathway (*n* = 100) and the medical led pathway (*n* = 100).

**Results:**

Both patient groups were found to have similar outcomes. In the clinical scientist led pathway 65% were discharged, 6% were found to have HF with reduced ejection fraction (HFrEF), 20% were found to have HF with preserved ejection fraction (HFpEF), 7% were referred into general cardiology, 1% were referred to the cardio-thoracic surgical team and 1% were admitted. In the medical led pathway, 61%, were discharged, 7% were found to have HFrEF, 23% were found to have HFpEF, 6% were referred into a general cardiology, 3% were referred to the cardio-thoracic surgical team and 0% were admitted. A significant reduction (30.1 days vs. 10.6 days) in time was observed from echocardiogram to outcome correspondence for patient. (*p* < 0.01).

**Conclusion:**

The scientist led pathway demonstrated improved time from diagnostic testing to outcome correspondence, ensuring timely enrolment on the relevant treatment pathway. Findings suggest that the pathway is feasible and effective, highlighting the need to utilise expertise of clinical scientists in the HF paradigm. Future work should explore long-term feasibility and sustainability of these clinics, further expansion and the feasibility of this service in a district general hospital or community diagnostic centres.

## Introduction

Heart failure (HF) is a progressive clinical syndrome resulting from an abnormality in cardiac structure/function that causes inadequate cardiac output secondary to increased left ventricular filling pressure [1]. This collection of physiological abnormalities, typically detected by transthoracic echocardiography (TTE), coupled with clinical symptoms/signs and an elevated natriuretic peptide level facilitate the diagnosis of the clinical syndrome of HF.

### HF in the UK

HF is highly prevalent in the United Kingdom (UK), with the PULSE study finding a prevalence of 1.9% in the adult population [2]. The ATLAS study revealed a UK median annual incidence of 3.3–3.5 per 1000 people [3] and this incidence increases with age [2].

The clinical syndrome is progressive in nature, with sequential phases from subtle initial symptoms on exertion, to marked symptom burden at rest [4]. In context of the progressive and persistent nature of the disease, clinical events are common, with 63,530 confirmed hospitalisations in England and Wales in 2022/23 [5]. Not surprisingly, it is an expensive condition to manage, with an estimated 5-year cumulative cost of £12,500 per patient [5].

Early identification and diagnosis of the disease is therefore critical in achieving the best outcomes for the patient, with diagnosis within the acute setting being associated with worse outcomes and higher mortality [6]. Prompt treatment with well-established and proven therapies [7] has been repeatedly shown in trial data to improve symptoms and quality of life as well as a reduce the risk of all cause death or HF related hospital admissions [8–10].

Current UK National Institute for Clinical Excellence (NICE) guidelines [11] recognise the importance of HF as a disabling clinical condition and promote prompt HF diagnosis from symptoms, signs and investigations. This includes taking a patient’s history, performing a clinical examination and blood chemistry measurement of N-terminal pro-B-type natriuretic peptide (NT-proBNP) levels. Those with a NT-proBNP level above 2,000 ng/L should be urgently referred for specialist assessment and TTE within 2 weeks and for individuals with NT-proBNP levels between 400 and 2000 ng/L, specialist assessment and TTE should occur within 6 weeks.

Referral into outpatient pathways are usually made via their General Practitioner (GP) in the UK to secondary care specialist teams and placed on “urgent” (NT-proBNP > 2000 ng/L) and “non-urgent” (NT-proBNP 400–2000 ng/L) diagnostic pathways.

### Our service

The Royal Wolverhampton NHS Trust serves a secondary-care patient population of approximately 500,000 people. It is also one of four regional cardio-thoracic centres covering the West Midlands region of the UK. It has historically employed an “urgent” (NT-proBNP > 2000 ng/L) HF diagnosis pathway using a “one stop” model combining a consultant outpatient appointment and a TTE appointment allowing rapid diagnosis and initial appropriate treatment of HF (i.e. initiation of medication, heart rhythm management or surgical intervention). A second, “non-urgent” (NT-proBNP 400–2000 ng/L) HF diagnosis pathway, involves an outpatient TTE being performed and then a remote review and triage of the referral and TTE result by a HF consultant resulting in discharge if HF is not identified, an urgent outpatient HF clinic appointment if HF is present, or onward referral into relevant specialities (e.g. General Cardiology, Cardiothoracic surgery, Arrhythmia clinic, etc.).

### Potential pitfalls

There is considerable demand on both pathways with an average of 12 “non-urgent” referrals per week. The “non-urgent” pathway then requires coordination from a broad multidisciplinary team (MDT) to ensure that NICE guidelines are met. There are several handovers built in to both pathways that can impact the diagnosis and outcome time. The pathway is dependent on timely triage of the initial GP referral and physical request of a TTE, capacity within a TTE department to accommodate these scans as “soon” (i.e. 6 week) requests, timely completion of TTE reporting, review/clinical outcome/decision, correspondence dictation and then correspondence typing and distribution. Under the NICE guidelines all these steps need to be completed during the 6-week timeframe and any delay could impact patient prognosis and patient anxiety/apprehension of awaiting results [12].

Despite a general UK wide push for early TTE in appropriate patients [13], our medical led (ML) HF diagnosis pathway is still dependent on significant input from cardiology consultants and their support team, with time from primary care referral into secondary care highlighted as a factor in treatment delays for HF patients [14].

### Rationale for current project

Recent NHS workforce plans aim to utilise and develop non-medical roles [15] to take on more complex and involved tasks and responsibilities (i.e. advanced clinical practice (ACP) roles). This support has been welcomed by this group of healthcare professionals, with clinical scientists/cardiac physiologists undertaking further education in health assessment allowing a more holistic approach to patient care. Physiologist led valve clinics are now an established and recommended practice for the assessment and conservative management of valvular heart disease (VHD) [16, 17]. These models incorporate a one-stop clinic approach of TTE and clinical assessment to ensure a patient focused and more cost-effective method for follow up of VHD [16]. Initial investigations into both ACP and Pharmacist led HF clinics have also been conducted [18, 19].

### Purpose

Utilising similar components of these established pathways but within the HF paradigm, we, in close collaboration with our HF team, trialled a cardiac clinical scientist led (CSL) Heart Failure diagnosis clinic for non-urgent suspected HF referrals, aiming to produce a more streamlined pathway.

The purpose of this pilot was to evaluate the effectiveness and outcomes of a CSL clinic at the Royal Wolverhampton NHS Trust. Our aim was to ensure timely clinical assessment at the point of TTE and reduce delays between TTE results and outcome correspondence making for the patient cohort of our “non-urgent” pathway.

## Methods

The clinic was undertaken at the Heart and Lung centre of the Royal Wolverhampton NHS Trust. Patients were selected for the clinic during departmental triage of TTE referrals made by our HF team after an initial GP referral into our HF service. As a result of this alignment with the existing pathway, there was no implicit delay in care for patients through inclusion into this clinic. All selected patients were part of the “non-urgent” pathway and therefore with a NT-proBNP value between 400–2000 ng/l.

### Role of clinical scientist

A senior echocardiographer/cardiac physiologist with AHCS (Academy for Healthcare Science) registration, British Society of Echocardiography (BSE) Level 2 TTE accreditation, a Level 7 health assessment qualification and ASCST (Associate Member of the Society for Cardiological Science and Technology) Part 1 & 2 qualifications led the clinic, with HF consultant oversight. Clinics ran with 1-hour of allocated time per patient with 4 patients booked in per session in a cardiac investigations department clinic room with a TTE machine, Blood Pressure (BP) sphygmomanometer and electrocardiogram (ECG) machine available.

**Fig. 1:**
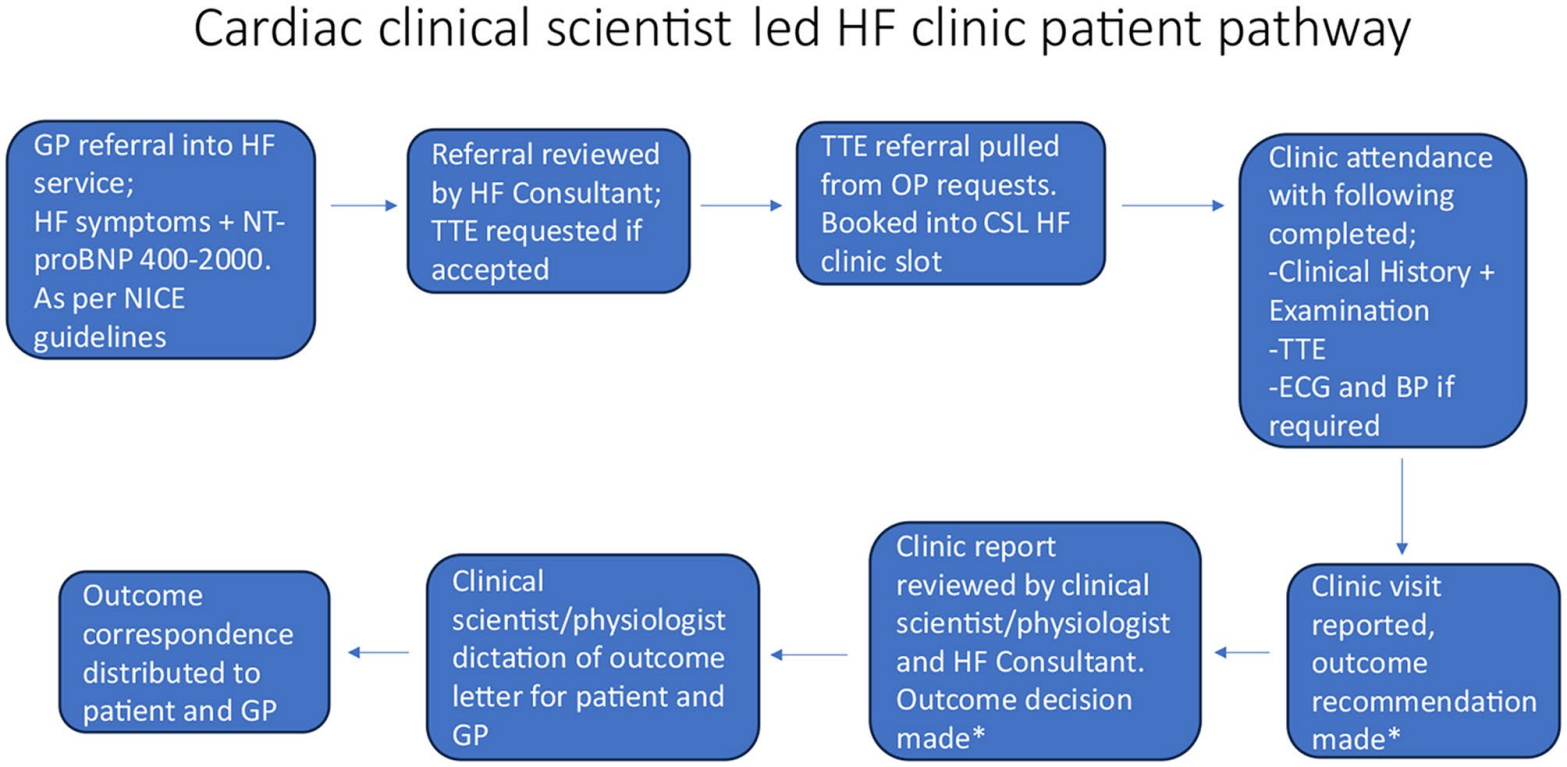
Cardiac clinical scientist Led HF clinic pathway. NT-pro BNP, N-terminal-pro B-type natriuretic peptide; ECG, electrocardiogram; GP, general practitioner; HF, heart failure; TTE, transthoracic echocardiogram; CSL HF, cardiac clinical scientist led heart failure pathway; MDT, multidisciplinary; NICE, National Institute for Heath and Clinical Excellence

Within the appointment a clinical history was taken, a full cardio-respiratory examination was performed, a standard BSE level 2 TTE, ECG and BP reading was also done (see Fig. 1 for clinic pathway).

**Fig. 2:**
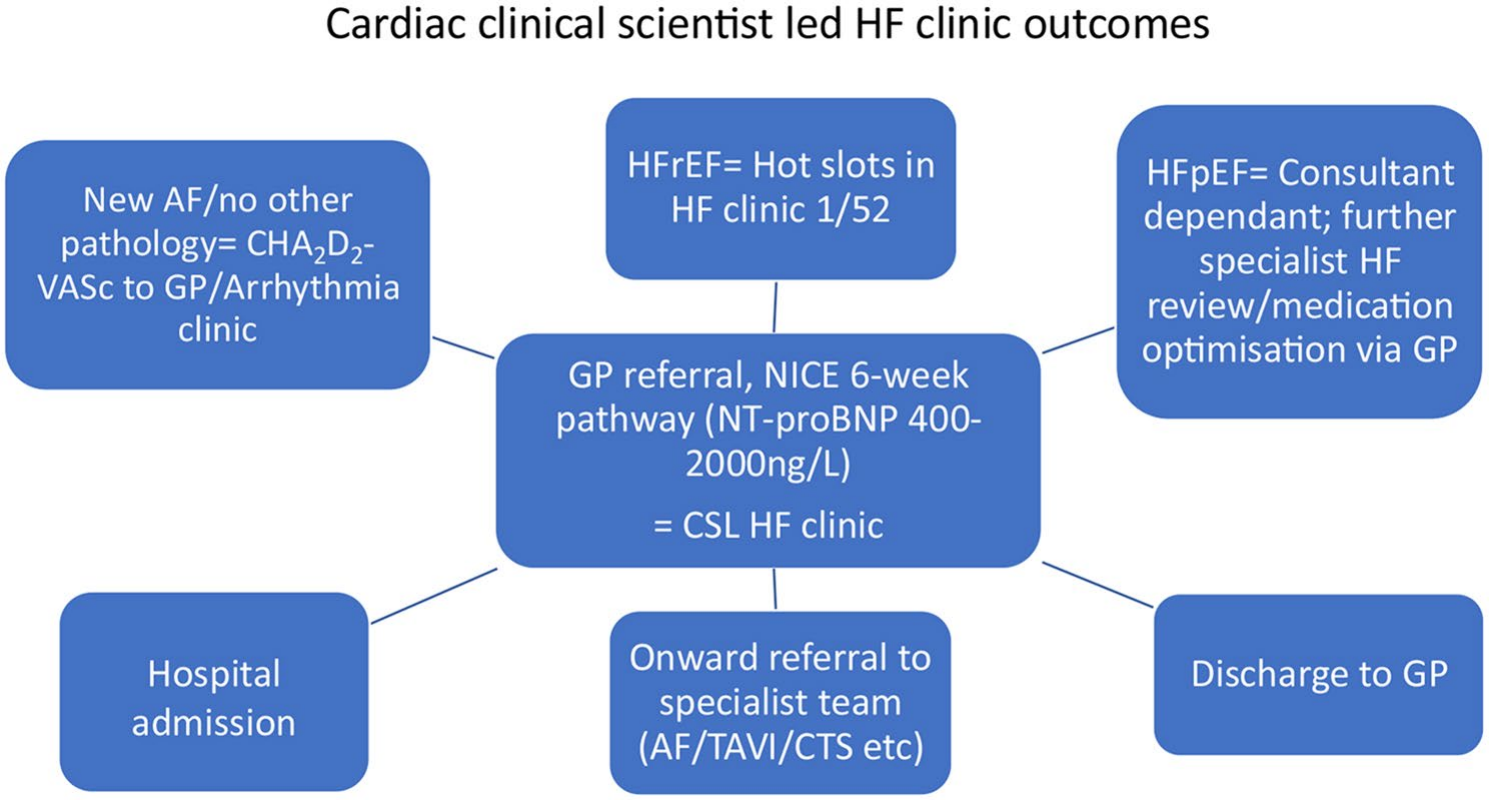
Cardiac clinical scientist led HF clinic outcome flow chart / MDT discussion. AF, atrial fibrillation; NT-proBNP, N-terminal-pro B-type natriuretic peptide; ECG, electrocardiogram; Echo, echocardiogram; Furo, furosemide; GP, general practitioner; HF, heart failure; HFpEF, heart failure with preserved ejection fraction HFrEF, heart failure with reduced ejection fraction; NICE, National Institute for Heath and Clinical Excellence; CSL HF, cardiac clinical scientist led heart failure pathway; TAVI, Transcatheter aortic valve implantation; CTS, Cardio-thoracic surgery

Post clinic, a full clinic report was created on our Phillips ISCV reporting system and a preliminary outcome recommendation made by the clinical scientist. These recommendations were typically that of the diagnosis of HFrEF (Heart Failure with reduced Ejection Fraction), suggestion of HFpEF (Heart Failure with preserved Ejection Fraction), there being no suggestion of HF or onward referral to a specialist team. Once the clinic had finished, cases were reviewed with a HF consultant, resulting in an outcome decision (see Fig. 2 for outcome flow chart). Correspondence letters were then dictated by the cardiac clinical scientist before being typed and distributed to both patient and referring GP by the Cardiac Investigations department secretary.

A retrospective review of 100 patients seen on each CSL and ML pathways was performed comparing predominantly clinical outcomes and TTE to correspondence time, with local approval for this service evaluation achieved. All clinical data was obtained from EHR for assessment of all clinical outcomes and timing of outcome correspondence. Data was obtained weekly over the period of 1 year.

### Statistical analysis

Continuous variables were initially assessed for normality using the Shapiro–Wilk test, which demonstrated that the ML pathway data were normally distributed, whereas the CSL pathway data were not. Consequently, normally distributed data are reported as mean (standard deviation), non-normally distributed continuous data as median (interquartile range), and categorical data as frequency (percentage). Owing to the small sample sizes observed, the Fisher–Freeman–Halton exact test was employed to compare clinical outcomes between pathways, and the Mann–Whitney U test was used to evaluate differences in TTE-to-outcome correspondence time.

## Results

The patient composition of CSL and ML cohorts were broadly similar. The CSL cohort consisted of 53 females and 47 males with a mean age of 78 (± 8.6 years) and a mean NT-proBNP value of 818ng/L (± 429). Within the ML cohort, 60 were female and 40 were male. The cohort had a mean age of 80 (± 8.5 years) and a mean NT-proBNP value 910ng/L (± 562) (see Table 1).

**Table 1:** ; patient demographics of both cardiac clinical scientist and medical led pathways

Patient Demographics				
	Cardiac clinical scientist-led pathway		Medical-led pathway	
Sample size	100		100	
Sex	Female 53 Male 47		Female 60 Male 40	
Age (years)	78 ± 8.6		80 ± 8.5	
BNP values (ng/L)	818 ± 429		910 ± 562	
Patient outcomes	Discharged HFrEF HFpEF General Cardiology CTS/ TAVI referral Hospital admission	65 6 20 7 1 1	Discharged HFrEF HFpEF General Cardiology CTS/ TAVI referral Hospital admission	61 7 23 6 3 0

Continuous data are presented as mean (± standard deviation) or median (interquartile range) and categorical data are presented as n (%)

CTS, cardiothoracic surgery; HFpEF, heart failure with preserved ejection fraction; HFrEF, heart failure with reduced ejection fraction; TAVI, transcatheter aortic valve implantation

### Clinical outcomes

Clinical outcomes with the CSL group showed that 65 patients were discharged back to their GP as a HF diagnosis had been ruled out, 6 patients clinic reports suggested a diagnosis of HFrEF and 20 suggested a diagnosis of HFpEF. 7 patients were referred on to General Cardiology (Gen Cardio) and 1 was referred to the Cardio-thoracic surgical team/TAVI team (CTS/TAVI) with severe aortic stenosis. A single patient was admitted due to the identification of aortic valve infective endocarditis.

Within the ML group, clinical outcomes showed that 61 patients were discharged back to their GP as a HF diagnosis had been ruled out, 7 patients were found to have HFrEF and 23 a diagnosis of HFpEF. 6 patients were referred on to General Cardiology and 3 were referred to the Cardio-thoracic surgical team/TAVI team.

**Fig. 3:**
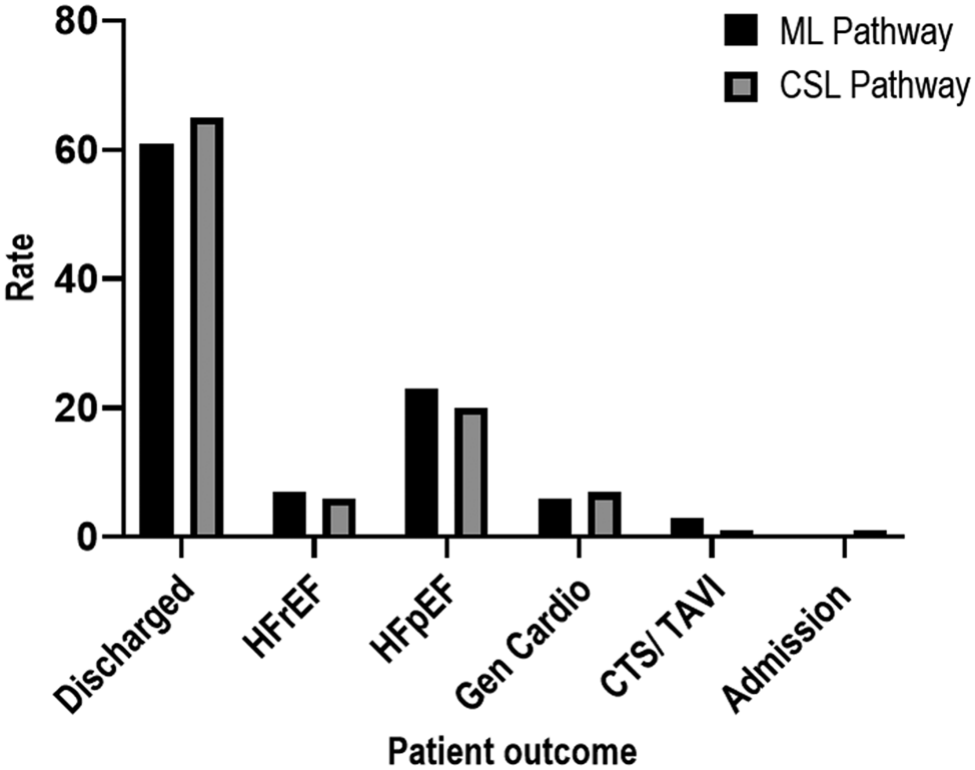
Patient outcomes from both ML and CSL pathways. HFpEF, heart failure with preserved ejection fraction HFrEF, heart failure with reduced ejection fraction; CSL, cardiac clinical scientist led pathway; ML, medical led pathway; TAVI, Transcatheter aortic valve implantation; CTS, Cardio-thoracic surgery; Gen Cardio, General Cardiology

Clinical outcome comparison is represented in Fig. 3. Differences in clinical outcomes between pathways were not statistically significant (Fisher-Freeman-Halton-Exact test, *p* = 0.836).

### TTE to correspondence time

**Fig. 4:**
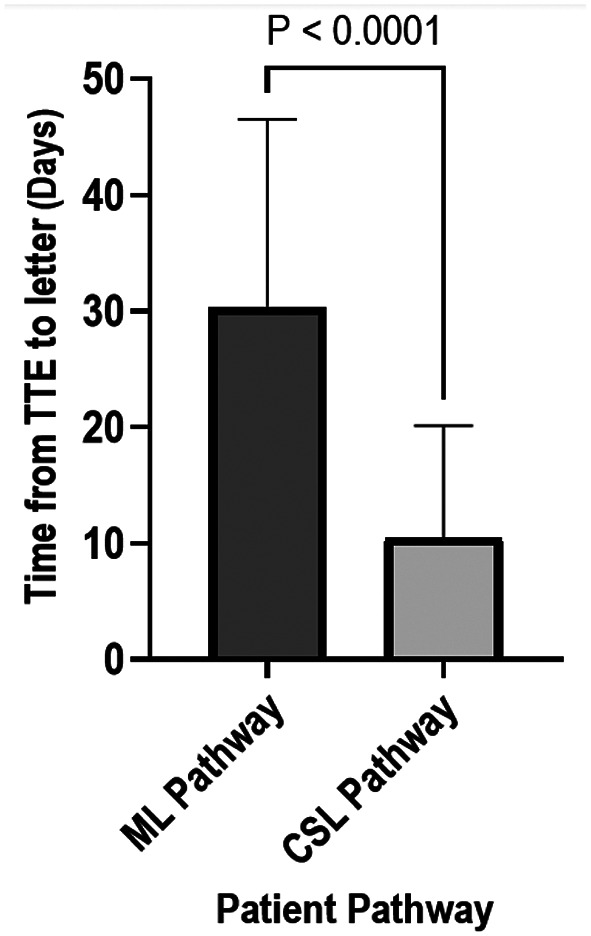
Time between TTE and outcome correspondence on ML and CSL pathways. CSL, cardiac clinical scientist led; ML, Medical led; TTE, transthoracic echocardiogram

Time from TTE to outcome correspondence was significantly reduced in the CSL pathway in comparison to the ML pathway (10.6 (IQR) days vs. 30.1 (IQR) days, respectively; *p* < 0.01). See Fig. 4.

## Discussion

Compared to the existing ML HF diagnosis pathway, our pilot CSL HF diagnosis pathway for patients requiring non-urgent review for suspected HF following NICE guidance, was found to be similarly effective delivering clinical outcomes and onward management for the patients referred and reduced time from assessment to outcome correspondence, permitting more timely treatment initiation.

### Cardiac clinical scientist viewpoint

From a cardiac clinical scientist perspective, this novel model delivering patient care allows an avenue for progression and development of the role, increasing autonomy and responsibility with sufficient safety netting in place. Although potentially daunting, the uncertainty of an assessment of a potentially complex patient with multiple comorbidities (providing sufficient support is given) will yield invaluable experiences, contributing to a more rounded, holistic practitioner, who is armed with the skills and knowledge that can be easily transferable to other CSL services. The unique skill set and experiences of a cardiac clinical scientist also stands them in good stead to answer the specific clinical question of “query HF”. The demands and requirements of this model also fulfils NHS plans for the utilisation of non-medic roles as previously outlined [15].

The model of a CSL HF diagnosis clinic also allows the further integration and collaboration of all disciplines involved in HF diagnosis and management, solidifying and developing the MDT that is needed for effective HF management, and it is only recently that clinical scientist/physiologist input into MDT and clinical guidance has begun to be recommended [20]. Time and interaction with HF consultants and nurses provide invaluable learning for cardiac clinical scientists and vice versa; communication that can only benefit the patient. A medical advocate is therefore paramount to facilitate, support and promote this pathway model and the role of the clinical scientist.

Considering the broader topic of profession development, this clinic pathway can hopefully provide a viable opportunity for role progression, particularly with junior/training staff currently specialising in TTE typically earlier on in their careers than previous.

This adjustment in patient care was undertaken with no additional funding. Consequently, there were difficulties encountered during the process of setting up the clinic, significant coordination and collaboration was needed from several large teams already involved in the care of these patients. Agreement of a process and protocol between HF consultants, HF administration, Imaging consultants, TTE department and Cardiac Investigations administrative teams was difficult to coordinate. Logistical issues were also encountered with coordination of administrative teams, lack of integrated referral, reporting, dictation and electronic records systems as well as access to primary care records. Considerable efforts were needed to ensure completeness and sufficient closure of the care episode.

The complexity and potential comorbidities present in a HF patient commonly attending a cardiology secondary care appointment for the first time has a level of uncertainty. It is therefore essential that cardiac clinical scientists working within this pathway have significant previous experience of a variety of scenarios to be equipped with the skill set needed to deal with a degree of unpredictability (see Governance considerations section below).

The increased interaction and narrative with patients during the clinic felt well received, there was a feeling of reassurance within the patients, knowing that answers were being sought in the context of a poorly understood blood test result and that any necessary action could be taken urgently or in good time.

### Other considerations

#### Cost

This pathway can streamline care of query HF GP referrals, reducing administrative and consultant time needed in typically already understaffed NHS departments. Although clinic appointments are 15 min longer than our current TTE appointment slots, increased involvement from the cardiac clinical scientist and reduced involvement of consultants allows a significant cost saving. Typically, consultant’s annual salaries will be double that of a band 8a cardiac clinical scientist [21]. Design of the clinic also fulfils NHS England’s recommendation to upskill its current workforce, contributing to further cost savings [14, 22].

#### Governance

The clinic has a dedicated Standard Operating Procedure (SOP) that has been reviewed and presented at a Cardiology Clinical Governance meeting. Included within this SOP are Audit templates adopted from previous physiologist led Valve clinic SOPs and patient satisfaction questionnaires.

It is paramount that cardiac clinical scientists working in this clinic have the required qualifications and experience, the clinics SOP includes an internal sign off package and below is a suggested table of “essential” and “desirable” criteria we feel appropriate to the clinic (Table 2). Timescales of individual training will vary from centre to centre and service provision. My personal experience has included 4 years of performing clinical assessments in consultant led valve clinics, performing TTE’s within the “one stop” urgent HF pathway (NICE 2-week, NT-proBNP > 2000ng/L) as well as the below criteria. The increasing number of clinical scientist/physiologist led services and potential exposure to them for individuals may well enable the process to be streamlined and shortened.

**Table 2:** Recommended skill set and criteria for cardiac clinical scientist/specialised cardiac physiologist (Heart failure Clinic)

Recommended skill set and criteria for specialist heart failure cardiac physiologist/clinical scientist	
Essential	Desirable
Level 2 TTE Accreditation	3-year post TTE Accreditation experience
AHCS/HCPC registration (i.e. STP Equivalence)	Highly developed practical experience
Level 7 Heath Assessment Qualification	Previous experience in physiologist led services
SCST Diploma in ECG interpretation (or equivalent)	Experience of shadowing consultant clinics
Dexterity and knowledge of a range of diagnostic equipment (ECG and BP)	
Experience of performing clinical assessments	

AHCS, Academy for Heathcare Science; HCPC, Heath and Care Professions Council; TTE, transthoracic echocardiogram; SCST, The Society for Cardiac Science and Technology; STP, scientist training programme

This pathway is reliant on consultant overview allowing an added level of security and safety. Clinics are currently ran alongside a HF outpatient clinic, with a supervising HF consultant available for urgent queries or advice, there is also a reliance on the availability of a HF consultant for mini-MDT/review of clinic patients.

### Limitations

Analysis of this clinic has shown positive impacts on the time taken to assess the patient and produce an outcome. However, the pathway may be limited and difficult to implement. Pathway set up is dependent on close collaboration of several departments and infrastructures as well as being dependant on HF consultant input. This is a single centre experience and translation into other centres may require different considerations. Service set up would require individual service evaluation to adopt this type of pathway structure.

Our initial study had a limited cohort size of 100 patients. Accordingly, although our proposed pathway is shown to be effective and efficient, analysis of a larger sized cohort would be more powerful. Although there was a general feeling within the clinic that the pathway was well received by patients, formal investigation into patient satisfaction would also give valuable insight into the pathway’s reception.

Data for this clinic has been collected over the period of one year, assessment of longer-term outcomes would contribute to the risk management of the clinic. Analysis of data such as re-referral rate, occurrence of hospitalisation with HF in the following 12 months and 1 year survival rate will allow further understanding of the clinic. There is the possibility that, with longitudinal data, negative outcomes/endpoints may be associated with a longer scan to referral time seen in the ML pathway.

NICE guidelines [11] state a 6-week time frame for this clinics cohort to receive a TTE and expert assessment, this clinic has been designed to allow that target to be met but may need to adapt to updated and new guidance, particularly in light of recent focus on the diagnosis and treatment of HFpEF.

### Future development

This limited pilot of a CSL HF diagnosis clinic has numerous possibilities for future development and expansion. A larger data set and patient database with recordings of symptoms, NYHA class and comorbidities for example, would allow greater detail of the patient cohort and may yield sub-groups of higher and lower risk referrals dependant on the prevalence of HF in certain patient types.

4 patients were booked into a single clinic with during 1 session per week. Current referral rates of “non-urgent” (NT-pro BNP 400-200ng/L) patients within our trust are around 12 per week. Therefore, there is scope for multiple clinics to be carried out during a week, compounding the benefits of reduced TTE to outcome time and reduced consultant input time. Expansion would obviously be dependent on staff and equipment availability but also the additional administration burden needs to be factored into departmental administrative staff job plans.

There are numerous avenues that can be utilised to not only expand the scope of this pathway but also increase the autonomy of cardiac clinical scientists and other practitioners. Although not readily available and common at this moment in time for cardiac clinical scientists and physiologists, the utilisation of prescribers such as HF pharmacists or nurse independent prescribers within the clinic would allow rapid initiation and up titration of medication [7].

With continued exposure and experience of the clinic, consultant involvement and reviews of initial outcome decisions made by the cardiac clinical scientist has the potential to be reduced further. As our data suggests, similar clinical outcomes were seen in both pathways, supporting the notion of increased cardiac clinical scientist autonomy whereby outcomes or for example, discharge outcomes are not reviewed by a consultant.

The feasibility of this clinic in differing settings should also be investigated. The pathway has the potential be applied in small district general hospitals or Community Diagnostic Centres in line with NHS plans [22].

## Conclusion

This new clinic pathway has illustrated a reduction in TTE to outcome correspondence as well as similar patient outcomes to our current ML pathway, streamlining the process of “query HF” referrals from primary care for patients requiring specialist assessment and TTE within 6 weeks. The pathway also has the potential to further expand the role of physiologists/clinical scientists as well as the HF MDT, allowing timely care and treatment of this patient cohort.

## Data Availability

The dataset used for this study are not available due to Information Governance restrictions. All data is presented.

## Abbreviations

ACP: Advanced Clinical Practitioner
AF: Atrial fibrillation
AHCS: Academy for healthcare science
ASCST: Associate member of the society for cardiological science and technology
BP: Blood pressure
BSE: British society of echocardiography
CSL: Cardiac clinical scientist led
CTS: Cardio-thoracic surgery
DM01: Diagnostic waiting times and activity
ECG: 12-lead electrocardiogram
GP: General Practitioner
HCPC: Health and care professions council
HF: Heart failure
HFpEF: Heart failure with preserved ejection fraction
HFrEF: Heart failure with reduced ejection fraction
MDT: Multi-disciplinary team
ML: Medical led
NHS: National health service
NICE: National institute for health and care excellence
NYHA: New York heart association
OSCE: Objective structured clinical examination
SCST: Society for cardiac science and technology
SOP: Standard operating procedure
STP: Scientist training programme
TAVI: Transcatheter aortic valve implantation
TTE: Transthoracic echocardiogram
UK: United Kingdom
VHD: Valve heart disease
